# Ultrasound of metacarpophalangeal joints is a sensitive and reliable endpoint for drug therapies in rheumatoid arthritis: results of a randomized, two-center placebo-controlled study

**DOI:** 10.1186/ar4034

**Published:** 2012-09-12

**Authors:** Matthew W Seymour, Stephen Kelly, Chan R Beals, Marie-Pierre Malice, James A Bolognese, Bernard J Dardzinski, Amy S Cheng, Corinne E Cummings, Steven S Smugar, Catherine McClinton, Amy Fox, William M Dooley, Constantino Pitzalis, Peter C Taylor

**Affiliations:** 1 Kennedy Institute of Rheumatology, Nuffield Department of Orthopaedics, Rheumatology and Musculoskeletal Sciences, University of Oxford, Windmill Road, Oxford, OX3 7LD, England; 2 John Vane Science Centre, William Harvey Research Institute, Centre for Experimental Medicine and Rheumatology, Barts and The London School of Medicine, Charterhouse Square, London, EC1M 6BQ, England; 3 Merck Sharp & Dohme Corp, One Merck Drive, Whitehouse Station, NJ 08889, USA; 4 Cytel, Inc., Cambridge, 675 Massachusetts Ave, Massachusetts, 02139, USA

## Abstract

**Introduction:**

We aimed to investigate the sensitivity and reliability of two-dimensional ultrasonographic endpoints at the metacarpophalageal joints (MCPJs) and their potential to provide an early and objective indication of a therapeutic response to treatment intervention in rheumatoid arthritis (RA).

**Methods:**

A randomized, double-blind, parallel-group, two-center, placebo-controlled trial investigated the effect on ultrasonographic measures of synovitis of repeat dose oral prednisone, 15mg or 7.5mg, each compared to placebo, in consecutive two-week studies; there were 18 subjects in a 1:1 ratio and 27 subjects in a 2:1 ratio, respectively. All subjects met the 1987 American College of Rheumatology criteria for the diagnosis of RA, were ≥18 years-old with RA disease duration ≥6 months, and had a Disease Activity Score 28 based on C-reactive protein (DAS28(CRP)) ≥3.2. Subjects underwent high-frequency (gray-scale) and power Doppler ultrasonography at Days 1 (baseline), 2, 8 and 15 in the dorsal transverse and longitudinal planes of all 10 MCPJs to obtain summated scores of quantitative and semi-quantitative measures of synovial thickness as well as vascularity. The primary endpoint was the summated score of power Doppler area measured quantitatively in all 10 MCPJs in the transverse plane at Day 15. Clinical efficacy was assessed at the same time points by DAS28(CRP).

**Results:**

All randomized subjects completed the trial. The comparison between daily 15 mg prednisone and placebo at Day 15 yielded a statistically significant treatment effect (effect size = 1.17, *P *= 0.013) in change from baseline in the primary endpoint, but borderline for prednisone 7.5 mg daily versus placebo (effect size = 0.61, *P *= 0.071). A significant treatment effect for DAS28(CRP) was only observed at Day 15 in the prednisone 15 mg group (effect size = 0.95, *P *= 0.032). However, significant treatment effects at all time points for a variety of ultrasound (US) endpoints were detected with both prednisone doses; the largest observed effect size = 2.33. Combining US endpoints with DAS28(CRP) improved the registration of significant treatment effects. The parallel scan inter-reader reliability of summated 10 MCPJ scores were good to excellent (ICC values >0.61) for the majority of US measures.

**Conclusions:**

Ultrasonography of MCPJs is an early, reliable indicator of therapeutic response in RA with potential to reduce patient numbers and length of trials designed to give preliminary indications of efficacy.

**Trial Registration:**

Clinicaltrials.gov identifier: NCT00746512

## Introduction

The development of new therapeutics for rheumatoid arthritis (RA) involves clinical assessment of response by endpoints that include composite measures of disease activity, such as the Disease Activity Score in 28 Joints (DAS28) [1], a continuous measure, and American College of Rheumatology (ACR) categorical responses [2,3]. Many of the component measurements are subjective, imprecise and insensitive to change and their use often necessitates lengthy and costly clinical trials using large cohorts of patients. This results in greater exposure to experimental drugs in early testing, many of which will eventually fail to receive approval.

For early testing of novel therapeutics, we require a sensitive method to distinguish between treatment groups in cohort studies that permits small patient numbers and provides a reliable, early indicator of efficacy. Ideally, such measures would be quick, non-invasive, objective, predict longer-term response to repeated medication and give an early indication of disease modification. Due to ethical constraints of performing placebo controlled trials and the resultant trend towards comparator controlled trials, the requirement for sensitive endpoints is greater than ever.

Metacarpophalangeal joints (MCPJs) are invariably involved in RA [4] and so their evaluation is important. These superficial joints are amenable to assessment with ultrasound (US) utilizing frequencies that produce high resolution images. High-frequency ultrasonography (HFUS) and power Doppler ultrasonography (PDUS) are reproducible tools for determining synovitis and more sensitive than clinical scoring in determining disease activity [5,6]. The synovial vascular signal on PDUS closely correlates with the dynamic contrast enhanced magnetic resonance imaging (MRI) in RA MCPJs [7,8] and synovitis detected by US predicts erosive disease [9-12].

By using a known efficacious treatment for RA, our objectives were:

1. To investigate the sensitivity and reliability of two-dimensional ultrasonographic endpoints (quantitative and semi-quantitative measures of synovial thickness and vascularity in MCPJs imaged in the dorsal longitudinal and transverse planes) and make comparisons between different endpoints. We have investigated the reliability of a summation of 10 MCPJs rather than the reliability on a joint by joint basis.

2. To determine the potential of two-dimensional ultrasonographic endpoints to provide an early and objective indication of a therapeutic response to treatment intervention in rheumatoid arthritis (RA).

3. To determine if there is a dose-response relationship between the two different relatively low, corticosteroid doses (15 mg and 7.5 mg) and ultrasonographic endpoints.

4. To compare the US endpoints with DAS28(CRP) (C-reactive protein) and to explore the potential of composite endpoints (DAS28 combined with US endpoints) to improve the registration of a significant treatment effect.

## Materials and methods

### Patients

Protocol 088 (clinicaltrials.gov identifier: NCT00746512) was a randomized, double-blind, parallel-group, placebo-controlled trial conducted at two academic research centers in the UK. Two panels were planned for the study: In Panel A, subjects were randomized in a 1:1 ratio to oral prednisone 15 mg daily or matching placebo for 15 days. After a total of 18 subjects completed the study, an interim analysis was planned to determine if this smaller sample size could significantly (alpha = 0.03, 1-sided) discriminate prednisone 15 mg from placebo based on the primary endpoint. If so, then enrollment in the prednisone 15 mg group would cease, and 27 additional subjects would be randomized (2:1 ratio) in Panel B to prednisone 7.5 mg or matching placebo for 15 days.

Two centers were chosen for feasibility of recruitment to the study. One of our general aims is to investigate the applicability of US endpoints in multi-center clinical trials.

Men and nonpregnant women ≥18 years old with RA for ≥ six months duration meeting the 1987 American College of Rheumatology criteria for the diagnosis of RA were eligible [13]. Subjects were required to have at least moderate disease activity (DAS28(CRP) ≥3.2) and moderate dorsal transverse synovial vascularity in two MCPJs (score ≥2) or severe in one MCPJ (score = 4) as measured on a semi-quantitative 0 to 4 scale.

Non-steroidal anti-inflammatory drugs (NSAIDs) at stable doses for ≥ four weeks were permitted, as were disease modifying anti-rheumatic drugs (DMARDs) at stable doses for ≥ six weeks, topical or inhaled glucocorticoids at stable doses for ≥ two weeks, and opiates at stable doses ≥ two weeks. Acetaminophen (paracetamol) was allowed for breakthrough pain, but NSAIDs were not to be taken on an as-needed basis.

Pertinent exclusion criteria included intra-articular glucocorticoid injections to MCPJs within three months or to non-MCP Js within six weeks of baseline; oral glucocorticoid use within four weeks; and current biological therapies.

Tolerability was assessed by clinical and laboratory examination and adverse event (AE) reporting during the study. After baseline measures subjects were randomized (by a sponsor statistician using a computerized Clinical Allocation Schedule System (CASS) with blocking factors to ensure blinding based on a multiple of the number of treatment groups and subjects) and then received their first dose of study medication.

All subjects gave informed written consent to participate. The study was conducted in accordance with the principles of Good Clinical Practice and approved by the institutional review board for human research.

### Ultrasonography

Assessment of reliability has been complicated by a diversity of nomenclature employed by different investigators. Terms for assessment of reliability are redefined below to avoid confusion. The ultrasonographer is usually also a reader of the anonymized images.

Within scan intra-reader: one patient, one ultrasonographer acquires one scan set, one reader reads the scan set twice; each reading is separated by a fixed time period (previously called intra-reader [14] and intraobserver [10,15]).

Within scan inter-reader: one patient, one ultrasonographer acquires one scan set, two independent readers (previously called inter-reader [14], interobserver [11,16-18] and inter-investigator [19]).

Parallel scan intra-reader: one patient, one ultrasonographer acquires two scan sets sequentially, one reader independently reads both scan sets (previously called intraobserver [20]).

Parallel scan inter-reader: one patient, two ultrasonographers each acquire a scan set independently, two readers each read their own acquired scan set independently (previously called interobserver [15,16,20-26]).

Imaging was performed at two centers (Kennedy Institute of Rheumatology (KIR) and St Bartholomew's and the London National Health Services Trust (B&L)) by two ultrasonographers (MS and SK, each with more than two years experience), both blinded to the subjects' group allocation. They spent approximately 16 hours together before the study to gain consensus on image acquisition and analysis. To determine reliability (within scan inter-reader, parallel scan intra-reader and parallel scan inter-reader) three scans were sequentially acquired at Day 1 (baseline) and on Day 15 according to Table 1.

**Table 1:** Scan/Reader Scheme.

Site	Scan	Ultrasonographer	Reader
1 (KIR)	1	1 (MS)	1 (MS)
			2 (SK)
	2	2 (SK)	2
	3	1	1
2 (B&L)	1	2 (SK)	2 (SK)
			1 (MS)
	2	1 (MS)	1
	3	2	2

Within scan inter-reader reproducibility is obtained by comparing scan 1values across the two readers. Parallel scan intra-reader reproducibility is obtained by comparing scan 1 read by the first reader and scan 3 read by the first reader. Parallel scan inter-reader reproducibility is obtained by comparing scan 1 read by the first reader and scan 2 read by the second reader. The scans acquired and read by the site ultrasonographer-reader (indicated in italics) were used to calculate treatment effects. KIR: Kennedy Institute of Rheumatology and B&L: St Bartholomew's and the London NHS Trust. MS based at KIR travelled to B&L for the subjects' Day 1 and Day 15 imaging and vice versa for SK who was based at B&L.

Using a GE Logiq9 ultrasound machine with a two-dimensional M12L transducer at each center, subjects underwent HFUS and PDUS scanning over the dorsum of all 10 MCPJs at Days 1, 2, 8 and 15 in the longitudinal and transverse (over the triangular structure - method previously described [27]) planes. Settings were identical on both GE Logiq9 ultrasound machines: HFUS (gray-scale) - Frequency 14 MHz; PD - Frequency 7.5 MHz, Gain 41, PRF 1.4 kHz, Wall Filter 127 Hz. With a view to standardization of data acquisition, the hands were maintained in a position of rest by a splint (identical at both sites). The time of day of scanning at each visit was within 1 hour of the time of the baseline visit. Care was taken when scanning to avoid undue pressure with the probe in case this altered blood flow in the joint. This was achieved by maintaining a distance of at least 1 mm of gel between the probe and the subject as visualized on the US monitor.

Stored clips and images were anonymized before reading. Each PDUS scan consisted of a three second movie clip. PDUS measures were made on the image frame at the peak of the PDUS signal and synovial area measures were made from the first technically qualified image on HFUS imaging.

**Figure 1:**
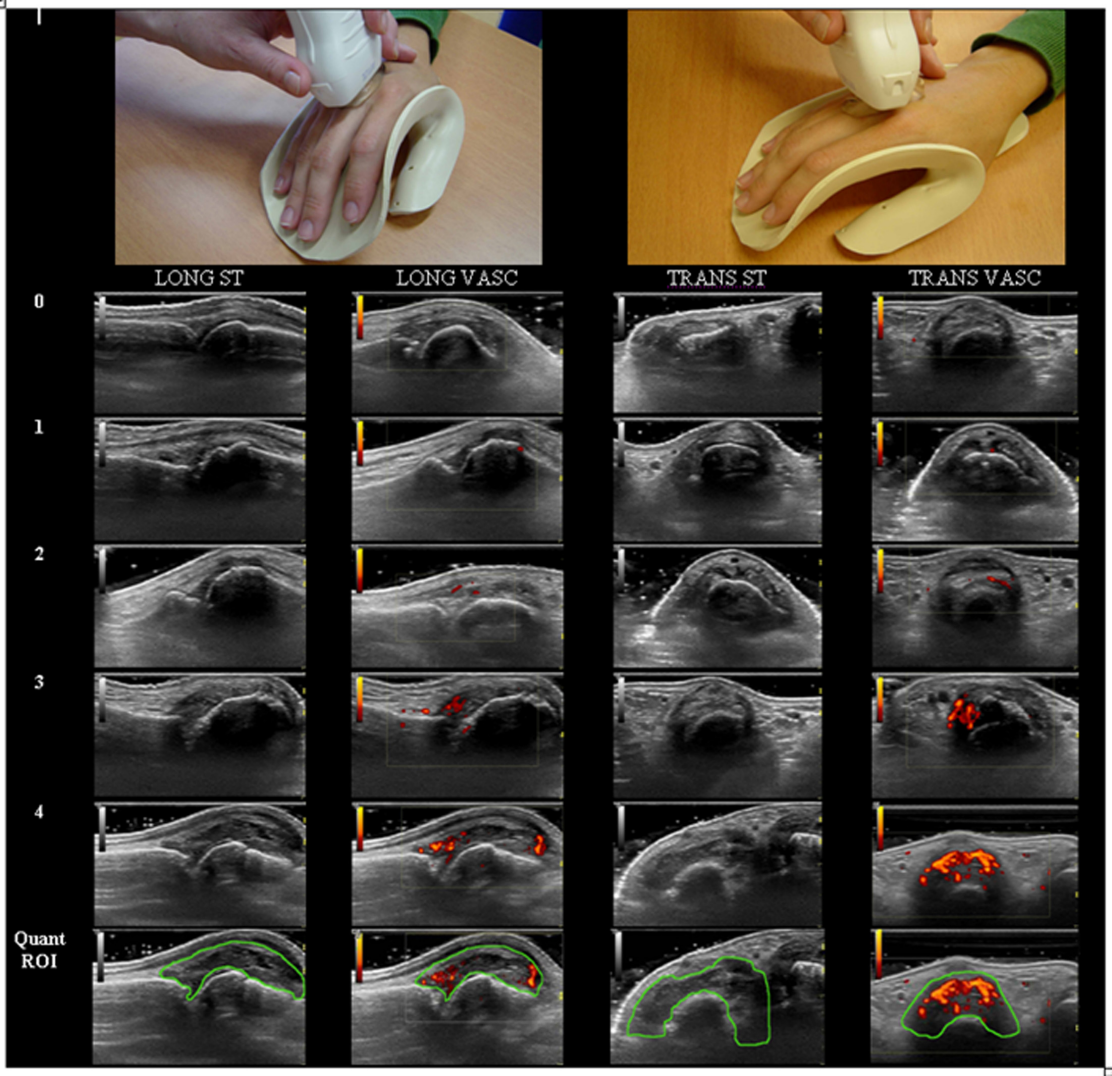
**Illustration of ultrasonographic scanning in the longitudinal and transverse plane using a splint to standardize image acquisition**. The four columns contain the semi-quantitative scales with scores from 0 to 4: 0 representing the lack of PD signal and 4 being severe PD signal; 0 representing no synovial thickening and 4 being severe synovial thickening. The last row demonstrates the region of interest (ROI) for quantitative analysis. Images were cropped for clarity. LONG: longitudinal, TRANS: transverse, ST: synovial thickening, VASC: vascularity, Quant ROI: region of interest for quantitative analysis, STA: synovial thickness area, PDA: power Doppler area.

The Synovial Thickness Area (STA), a quantitative measure, is a count of the number of pixels within a defined region of interest (ROI) in a standardized two-dimensional image of the joint. For the longitudinal STA (Long STA) the ROI should envelop the synovium over the phalangeal base, triangular structure, metacarpal head and metacarpal notch to the joint capsule superiorly. For the transverse STA (Trans STA) the ROI should envelop the MCPJ synovium from the lower border of the triangular structure (if bone, this is indicated by a continuous hyperechoic line or if cartilage by a homogenous anechoic line above bone) to the joint capsule superiorly (Figure 1). The transverse and longitudinal STA from each of the 10 MCPJs were summated in each respective plane to create the 10MCP Trans STA and 10MCP Long STA.

Synovial thickness (ST) was graded semi-quantitatively in each MCP joint against a standardized image set on an ordinal scale ranging from 0 to 4: 0, no synovial thickening; 1, minimal; 2; mild; 3, moderate; 4, severe (Figure 1). The longitudinal and the transverse ROIs that were compared with the representative images were the same as the respective STA ROIs. The saved gray-scale image was compared with the library and MS and SK decided which representative image was the closest fit with regard to area of ST and allocated a score. The scores from each of the 10 MCPJs were summated to create a Synovial Thickness Index (STi; minimum score of 0 and a maximum of 40) for each plane; the 10MCP Trans STi and 10MCP Long STi.

The Power Doppler Area (PDA), a quantitative measure, is a count of the number of pixels with PDUS signal, uncorrected for pixel intensity, within a defined ROI in a standardized two-dimensional image of the joint. The ROIs for longitudinal and transverse PDA are the same as the corresponding ROI for STA and therefore extraarticular digital vessels are excluded. If present, reflection artifacts from digital vessels are also excluded if they enter the ROI (Figure 1). The transverse and longitudinal PDAs from each of the 10 MCPJs were summated in each respective plane to create the 10MCP Trans PDA and 10MCP Long PDA.

PDUS was also graded in each MCPJ using a semi-quantitative 0-to-4 vascularity scale: 0, no PD signal; 1, minimal; 2; mild; 3, moderate; 4, severe. As for the PDA the longitudinal and the transverse ROIs were the same as the respective STA ROIs. Images were graded against a library of representative images (Figure 1), that is, for each selected image MS and SK visually estimated the amount of colored pixels within the joint capsule, compared this with the library, decided which representative image was the closest fit and allocated a score. The scores from each of the 10 MCPJs were summated to create a Vascularity Index (VASCi: minimum score of 0 and a maximum of 40) for each plane, the 10MCP Trans VASCi and 10MCP Long VASCi. The 10MCP Trans PDA was the primary endpoint; the other US endpoints were secondary. The transverse view was chosen as this had previously demonstrated its utility in differentiating two groups in a randomized placebo controlled trial [12].

Quantitative vascularity and ST measurements were analyzed using the free downloadable software program ImageJ version 1.41 with an in-house plugin written by BD which enabled a rapid review of each PD clip to find the frame that displayed the most activity.

Several published four point scales for power Doppler (PD) have no PD signal as the lowest grade (normal), presence of a single vessel (mild) as the next grade and then less than 50% (moderate) and greater than 50% (severe) PD signal to gray-scale signal within the ROI to determine the next two grades [6,21,26]. The majority of published four-point gray-scale semi-quantitative scales of synovial thickening/synovitis are explained simply as subjective grading with four points (normal, mild, moderate and severe) [6,21,23]. Those that are described in detail examine the extent at which synovitis breaches boundaries to determine the grade of synovitis [19,26]. Ordinal grading scales typically infer linearity and equidistance between grading points, whereas the above mentioned four-point vascularity scales lack grading points at low levels of Doppler signal, potentially underestimating change (either up or down) in response to therapy. Our rationale for increasing the semi-quantitative scales for scoring by one point was to provide semi-quantitative ultrasonographic outcome measures that are potentially more sensitive to change. We developed a five point scale for synovial vascularity. The basis for this is that within the above mentioned four-point vascularity scales there is potentially a leap between mild and moderate. In our experience there are often images that the four point scales [6,21,26] would class as moderate which we would still consider as mild, for example, two vessels and up to three small areas of confluence. We have made provision for this. On our scale these two examples would score two and, therefore, one vessel scores one on our scale, minimal. This additional grade may serve to improve the sensitivity. Therefore, another objective of this study was to investigate this five-point scale for vascularity and also the novel five-point scale for ST. In support of the library of images approach to semi-quantitative scoring of synovitis, a recent study that utilized a US atlas has reported excellent parallel scan inter-reader reliability (intra-class correlation coefficients (ICC) values: gray-scale 0.95 and PD 0.97) [28].

#### Clinical efficacy assessments

Clinical efficacy was assessed by the DAS28(CRP), which includes the number of swollen and tender joints (28-joint count), a patient's global assessment of arthritis index (visual analogue scale) and CRP. Assessments were performed by a single rheumatology research nurse in each center, independent of the US examinations. Each study nurse attended a DAS28 standardization training course within the preceding year of the study start.

#### Statistical analysis

The primary hypothesis for both panels was that prednisone (15 mg or 7.5 mg) would have a greater change from baseline in 10MCP Trans PDA (the primary endpoint) after 15 days of treatment. The analysis was performed using an analysis of covariance model with panel and treatment nested within panel as factors, and baseline value included as a covariate. Only observed data were analyzed; missing data were not imputed. The analyses were carried out for change from baseline at each of days 15, 8, and 2. Interpretation of *P*-value testing for each endpoint was made in a step-down fashion, in that order, at α = 0.05 (1-sided) for the primary endpoint. For Panel A Days 1 and 15, the first scan set acquired and read by the ultrasonographer-reader associated with the clinical site was used for analysis of treatment effects and for correlation with other endpoints; there was one scan set per visit for all other study time points.

For the interim analysis following Panel A, if the true underlying effect size for the primary endpoint was 1.0, the overall power, accounting for the interim and a potential final analysis if the study continued as originally planned, was approximately 88% for 18 subjects per group and approximately 83% for 15 subjects per group. The probability of stopping at the interim analysis was approximately 63%. These computations employ the Hwang, Shih, deCani gamma = 1 stopping criteria which yields α = 0.03, 1-sided at the interim and, if applicable, final analyses. This controls the overall alpha level at 0.05, 1-sided. If the study continued after the interim analysis, a sample size of 15 subjects per group had 80% power to detect a statistically significant (α = 0.05, 1-tailed) difference between prednisone and placebo assuming an effect size of 0.93.

Significance for the effect of the secondary imaging endpoints and DAS28(CRP) was not error-protected from chance significance associated with multiple comparisons. The rationale for this choice is to minimize the chances of false negatives because of the exploratory nature of these secondary endpoint analyses. Thus, they are viewed as hypothesis generating rather than conclusive.

The repeated measures on Days 1 and 15 of Panel A were used to assess reproducibility (Table 1) using intra-class correlation coefficients for each endpoint. Pearson correlation coefficients were computed among pairs of endpoints, including DAS components, with US endpoints, to assess association among them.

For the analysis of creating a composite PDUS and DAS28 endpoint, precision was measured by effect size (average effect of the 15 mg and 7.5 mg prednisone effects minus placebo divided by pooled standard deviation) using a model with panel and treatment nested within panel as factors. The method for building composites was the O'Brien global statistic which is the average of the standard z-scores across the endpoints.

All tests were performed one-sided at level 0.05.

## Results

### Patients

Baseline subject and disease characteristics are shown in Table 2. Due to an error in the randomization code, there was uneven subject allocation in Panel A. There were no significant differences between baseline characteristics in subjects enrolled in Panels A and B or between those in each treatment assignment within Panel A and B. All treatments were generally well-tolerated. A total of 19 subjects reported 31 AEs (including 6 AEs with onset at prestudy/pre-treatment). There was one serious AE of breast carcinoma which was not considered by the investigator to be drug-related. None of the subjects enrolled discontinued due to an AE.

**Table 2:** Baseline patient characteristics.

	Panel A		Panel B	
	**Prednisone 15 mg Number = 8**	**Placebo Number = 10**	**Prednisone 7.5 mg Number = 18**	**Placebo Number = 9**

Gender, number (%)				
Female	6 (75.0)	5 (50.0)	12 (66.7)	6 (66.7)
Male	2 (25.0)	5 (50.0)	6 (33.3)	3 (33.3)
Age, years				
Mean (SD)	61.6 (6.5)	58.4 (12.8)	57.9 (13.3)	55.1 (12.8)
Range	48 to 69	45 to 86	37 to 81	32 to 71
Race, number (%)				
Asian	0 (0.0)	3 (30.0)	2 (11.1)	3 (33.3)
Black	0 (0.0)	1 (10.0)	4 (22.1)	0 (0.0)
White	8 (100.0)	6 (60.0)	12 (66.7)	6 (66.7)
10MCP Trans PDA, mean (SD)	9.48 (6.44)	10.55 (9.54)	8.31 (8.66)	9.78 (7.28)
DAS28(CRP), mean (SD)	5.2 (1.3)	5.5 (1.6)	5.3 (1.2)	5.6 (1.8)
Number of tender joints, mean (SD)	10.9 (7.4)	13 (10.1)	13.6 (8.6)	18.4 (10.1)
Number of swollen joints, mean (SD)	10.1 (5.4)	12.5 (8.7)	11.3 (5.6)	16.3 (8.6)
CRP (mg/L), median (quartiles)	8.4 (4.2-25.3)	14.0 (3.5-37.2)	6.9 (1.7-14.6)	3.7 (0.7 - 11.4)
Patients with positive rheumatoid factor^a^, number (%)	6 (75.0)	6 (60.0)	16 (88.9)	6 (66.7)

In this instance, the 10MCP Trans PDA for Panel A used data from all three baseline scans. There were no statistically significant differences, *P *>0.05, between groups based on nonparametric Wilcoxon and t-tests. ^a^Positive rheumatoid factor was defined as rheumatoid factor >= 12 KU/L, the reference range for Quest assay. CRP, C-reactive protein; DAS28, Disease Activity Score in 28 Joints; MCP, metacarpophalangeal joint; PDA; Power Doppler Area; SD, standard deviation.

### Endpoint responsiveness

**Figure 2:**
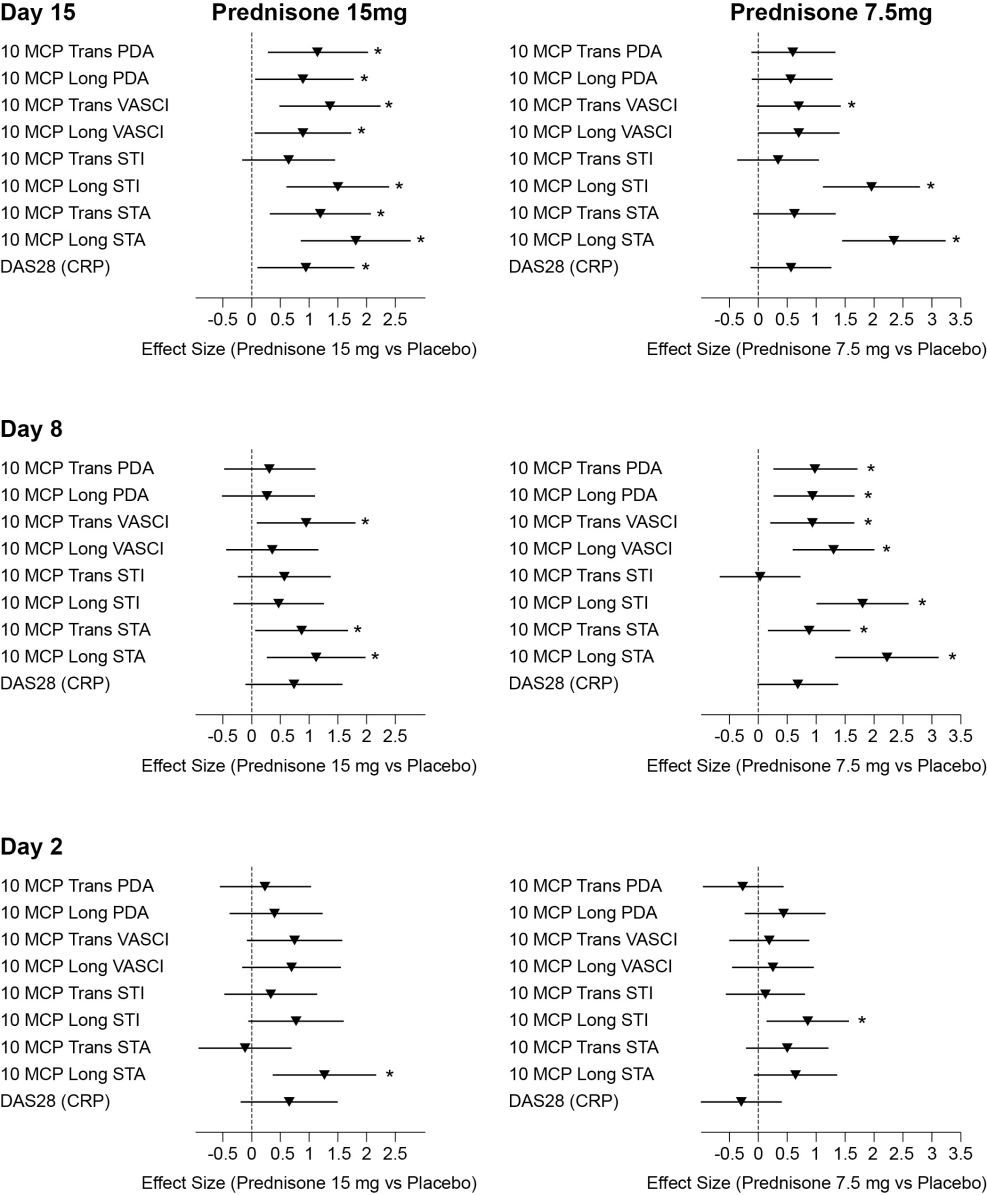
**Within-panel comparisons (panel A and B) of US and DAS28(CRP) endpoint responsiveness**. Effect size and 90% CI are presented. Power Doppler measures: PDA: Power Doppler Area; continuous measure of PD signal within the synovium; VASCi: Vascular Index (0 to 4 scale). Gray scale US measure of synovial swelling: STA: Synovial Thickness Area; area bounded by synovium; STi: Synovial Thickness Index (0 to 4 scale). ^* ^Significant effect: *P *<0.05. CI, confidence interval.

The comparison between prednisone 15 mg daily and placebo at Day 15 in Panel A yielded a statistically significant treatment effect (effect size = 1.17, *P *= 0.013) in change from baseline in the primary endpoint, 10 MCP Trans PDA, but not for Panel B (prednisone 7.5 mg daily versus placebo) (effect size = 0.61, *P *= 0.071). The primary endpoint was also met as early as Day 8 in Panel B with 7.5mg daily prednisone (effect size = 0.98, *P *= 0.01) but no statistically significant treatment effect was detected at Day 2 or at Days 2 and 8 in Panel A, 15mg prednisone daily (Figure 2).

A significant effect of prednisone 15 mg was observed for the secondary endpoint, DAS28(CRP), only at Day 15 (effect size = 0.95, *P *= 0.032). Prednisone 7.5 mg did not show any significant effect for DAS28(CRP) at any time point.

With respect to the seven secondary US endpoints, in Panel A there was a general trend for the number of significant treatment effects to increase from Day 2 to Day 15 (Figure 2). In Panel B the largest number of significant treatment effects was observed at Day 8. The US endpoint with the largest observed effect size, 2.33, was the 10MCP Long STA found within Panel B at Day 15. In fact, the effect size for this endpoint was numerically the largest at Day 15 and 8 (for both panels) and Day 2 (for Panel A); and was the only endpoint to demonstrate a significant treatment effect at Day 2 in Panel A. At Day 2 in Panel B, the 10MCP Long STi (a semi-quantitative rather than quantitative measure of longitudinal ST area) was the only endpoint to demonstrate a significant treatment effect. Another notable endpoint was the 10MCP Trans VASCi which demonstrated significant effect sizes in both Panels at Day 15 and 8 (largest effect size was within Panel A, Day 15 = 1.38).

None of the imaging endpoints showed a statistically significant difference between the treatment effects of the two prednisone doses. However, there was concordance in the rank ordering of effect sizes for US endpoints in keeping with the prednisone dose.

### Reproducibility of summated 10MCP score US endpoints

The within-scan inter-reader, parallel scan intra-reader and parallel scan inter-reader reliability were good to excellent for the majority of US measures. Overall mean ICC values for within-scan inter-reader, parallel scan intra-reader, and parallel scan inter-reader reliability were 0.77, 0.83 and 0.61, respectively. Overall mean ICC value for all quantitative measures of synovitis was greater than for all semi-quantitative measures, 0.77 and 0.70, respectively (*P *= 0.77). The overall mean ICC value for all longitudinal measures of synovitis was greater than for all transverse measures, 0.80 and 0.68, respectively (*P *= 0.16). The overall mean ICC value for all power Doppler measures of synovitis was greater than for all gray-scale measures, 0.84 and 0.63, respectively (*P *= 0.002). *P*-values were computed assuming independence of the ICC values via Wilcoxon Rank Sum test, and are two-sided.

The endpoints with the best agreement at baseline or on treatment were the VASCi (Long and Trans), both of which are semi-quantitative measures of vascularity but assessed in different planes (Table 3).

**Table 3:** Reproducibility of US endpoints.

10 MCP US endpoint	Reproducibility method	Baseline ICC (95% CI)	Day 15 ICC (95% CI)
Long PDA	Within-scan inter-reader	0.89 (0.80; 0.99)	0.98 (0.95; 1.00)
	Parallel scan intra-reader	0.80 (0.64; 0.96)	0.62 (0.36; 0.88)
	Parallel scan inter-reader	0.78 (0.59; 0.97)	0.68 (0.45; 0.92)
Long STA	Within-scan inter-reader	0.76(0.56; 0.95)	0.81 (0.65; 0.97)
	Parallel scan intra-reader	0.95 (0.90; 1.00)	0.83 0.68; 0.98)
	Parallel scan inter-reader	0.85 (0.72; 0.98)	0.71 (0.48; 0.95)
Long STi	Within-scan inter-reader	0.67 (0.40; 0.93)	0.54 (0.20; 0.88)
	Parallel scan intra-reader	0.87 (0.75; 0.99)	0.82 (0.66; 0.98)
	Parallel scan inter-reader	0.67 (0.42; 0.93)	0.46 (0.07; 0.85)
Long VASCi	Within-scan inter-reader	0.90 (0.81; 0.99)	0.94 (0.88; 1.00)
	Parallel scan intra-reader	0.93 (0.87; 0.99)	0.94 (0.88; 1.00)
	Parallel scan inter-reader	0.83 (0.67; 0.98)	0.85 (0.72; 0.99)
Trans PDA	Within-scan inter-reader	0.99 (0.98; 1.00)	1.00 (0.99; 1.00)
	Parallel scan intra-reader	0.75 (0.57; 0.93)	0.76 (0.57; 0.94)
	Parallel scan inter-reader	0.72 (0.51; 0.93)	0.75 (0.55; 0.96)
Trans STA	Within-scan inter-reader	0.52 (0.33; 0.72)	0.57 (0.36; 0.79)
	Parallel scan intra-reader	0.83 (0.68; 0.98)	0.92 (0.85; 0.99)
	Parallel scan inter-reader	0.57 (0.24; 0.89)	0.40 (0.01; 0.80)
Trans STi	Within-scan inter-reader	0.48 (0.19; 0.76)	0.36 (0.10; 0.62)
	Parallel scan intra-reader	0.83 (0.68; 0.98)	0.77 (0.58; 0.97)
	Parallel scan inter-reader	0.00 (-0.46; 0.46)	0.01 (-0.46; 0.48)
Trans VASCi	Within-scan inter-reader	0.92 (0.84; 0.99)	0.96 (0.92; 1.00)
	Parallel scan intra-reader	0.73 (0.52; 0.94)	0.91 (0.84; 0.99)
	Parallel scan inter-reader	0.68 (0.45; 0.92)	0.78 (0.60; 0.96)

ICC (intraclass correlation coefficient) values of <0.40, poor to fair agreement; 0.41 to 0.60, moderate agreement; 0.61 to 0.80, good agreement; and 0.81 to 1.00, excellent agreement. CI, confidence interval; PDA; Power Doppler Area; STA, synovial thickness area; STi, synovial thickness index; VASCi, vascularity index.

### Panel A correlations between US endpoints and DAS28(CRP)

Correlations assessed at baseline between US endpoints and DAS28(CRP) were moderate and ranged between 0.52 and 0.68; all were statistically significant (*P *<0.05). At Days 15, 8 and 2, 96% of the correlations in the placebo group were between 0.5 and 0.9 (actual range 0.23 to 0.9) and 92% of the correlations were between 0.4 and 0.8 (actual range 0.11 to 0.80) in the prednisone group.

### Exploratory composite endpoint responsiveness

Each composite endpoint is a simple sum of the standardized score corresponding to the DAS28(CRP) and two US endpoints, each summed across 10 MCPJs. Effect sizes at Days 15, 8 and 2 for composite endpoints constructed using the DAS28(CRP), Trans PDA/VASCi, and Long STA/STi in Panel A are shown in Table 4. These were selected as they generally had the largest effect sizes at the three post-treatment time points. All four composite endpoints demonstrated statistical significance after only a single day of dosing, that is, at Day 2.

**Table 4:** Performance of exploratory composite endpoints.

Endpoint	Scan	Day 15 Effect size (90% CI)	Day 8 Effect size (90% CI)	Day 2 Effect size (90% CI)
Z-score(DAS28+Trans VASCi+Long STi)	1	1.96 (0.97; 2.90)	1.41 (0.46; 2.32))	1.28 (0.40; 2.13)
Z-score(DAS28+Trans VASCi+Long STA)	1	2.05 (1.05; 3.01)	1.53 (0.56; 2.46)	1.14 (0.28; 1.98)
Z-score(DAS28+TransPDA +Long STA)	1	2.14 (1.12; 3.10)	1.17 (0.25; 2.05)	1.14 (0.28; 1.98)
Z-score(DAS28+TransPDA +Long STi)	1	1.93 (0.95; 2.87)	0.98 (0.09; 1.84)	0.95 (0.11; 1.76)
DAS28(CRP)		0.95 (0.11; 1.76)	0.75 (-0.12; 1.59)	0.67 (-0.15; 1.46)

Panel A (Prednisone 15mg versus Placebo) effect sizes for composite endpoints at Day 15, Day 8 and Day 2 compared with DAS28(CRP). The sum of the standardized score of DAS28(CRP), 10 MCP Trans VASCi, 10MCP Long STi (Z-score(DAS28+Trans VASCi+Long STi)); the sum of the standardized score of DAS28(CRP), 10 MCP Trans VASCi, 10 MCP Long STA (Z-score(DAS28+Trans VASCi+Long STA)); the sum of the standardized score of DAS28(CRP), 10 MCP Trans PDA, 10 MCP Long STA (Z-score(DAS28+Trans PDA+Long STA)); and the sum of the standardized score of DAS28(CRP), 10 MCP Trans PDA, 10 MCP Long STi (Z-score(DAS28+Trans PDA +Long STi)). Note: If the 90% confidence interval excludes zero (or values less than zero) then the value is significant at the <0.05 level for a one-sided comparison. We have prior information giving us the directionality of change and are only interested in change in one direction. Therefore, all the above effect sizes for composite endpoints were significant at all post dose time points and for DAS28(CRP) at Day 15 only. CI, confidence interval; CRP, C-reactive protein; DAS28, Disease Activity Score in 28 Joints; PDA; Power Doppler Area; STA, synovial thickness area; STi, synovial thickness index; VASCi, vascularity index.

The effect of prednisone 7.5 mg, Panel B, on the relatively easy-to-score pre-defined composite endpoint taken from the analysis of Panel A, Z-score(DAS28 + Trans VASCi + Long STi), was significant at Days 15 and Day 8 (effect sizes 1.84 and 1.80, respectively).

## Discussion

We have demonstrated that a wide range of HFUS measures of ST and PDUS measures of synovial vascularity at the MCPJs are reproducible and capable of detecting treatment effects of oral prednisone (15 mg and 7.5mg daily) after a week, and two US measures after only one day, in small panels of subjects (n = 18 and n = 27, respectively) with moderate to severely active RA. DAS28(CRP) was only able to detect a significant treatment effect after two weeks in the 15 mg cohort. US may, therefore, be a leading indicator of therapy response occurring before a clinical response. At present, more than 50% of drugs tested fail at phase III and the expense of the traditional drug development pathway has become prohibitive for numerous novel compounds developed to selectively inhibit a range of potential therapeutic targets that have been identified for RA. Our study has extensively investigated the sensitivity and reliability of a diverse range of two-dimensional ultrasonographic endpoints at the MCPJ and their potential as tools to provide an early and objective indication of a therapeutic response to treatment intervention in RA. We have confirmed that ultrasonography of MCPJ is an early, reliable indicator of therapeutic response in RA and it thus has the potential to reduce patient numbers required as well as the duration of clinical trials designed to give a preliminary indication of efficacy. Such an approach to early drug development in RA might increase the chances of success in later phase studies designed to meet the regulatory endpoints that are required to achieve approval.

In the present study, correlations between the majority of different US endpoints and DAS28(CRP) while on prednisone treatment were between 0.4 and 0.8, suggesting that they measure somewhat different constructs. Combining US endpoints with DAS28(CRP) increased effect sizes at all time points and identified treatment effects earlier. Composite endpoints increased the endpoint sensitivity for 15 mg in Panel A. The DAS28(CRP) had an effect size of about 1.0, which would take 13 subjects per group to identify a treatment difference (alpha 0.1; 80% power). In combination with a US endpoint with combined effect size of approximately 1.5, the sample number drops to six per group. Some combinations of US endpoints with effect sizes of approximately 2.0 would require four subjects per group. Likewise, single dose effects of 15 mg prednisone were identifiable with some combinations of endpoints. These findings strongly suggest a potential value in employing such composite endpoints in future prospective small studies designed to establish an early indication of efficacy. Composite endpoints were selected from Panel A on how well they performed. They were tested in Panel B in a predefined way but in a limited capacity. These composite endpoints need to be tested in future studies to confirm their utility.

Both 15 mg and 7.5 mg prednisone represent relatively low corticosteroid doses and it would be notable if an endpoint could differentiate their effect. Overall, there was a trend towards a dose-response. Greater numbers of subjects may have discriminated the two doses. Other factors that may have decreased the study's ability to differentiate the two doses include the fact that there were two centers, the scanning rooms of which, for example, may have been at different temperatures, and there were two ultrasonographers, the first scans of each in their respective centers were used to determine treatment effect.

For Panel B the 10MCP Trans PDA demonstrated a significant treatment effect earlier than in Panel A. This may be because there were more subjects in Panel B who received active treatment, albeit at a lower dose. To support this, at Day 8 more US endpoints registered a significant treatment effect for Panel B than Panel A.

For Panel A, seven out of eight US endpoints demonstrated a consistent time-response to 15mg of prednisone. Within Panel B more US endpoints registered a significant effect size at Day 8 in comparison with Day 15 perhaps due to waning of therapeutic response to low dose corticosteroid in some subjects. The observed transient response of the US endpoints to 7.5 mg of prednisone was mirrored in the effect sizes of the DAS28(CRP) even though this latter endpoint did not show significance at any time point. We postulate that for some subjects in Panel B, 7.5mg of prednisone may be just below the threshold dose for a sustained anti-inflammatory effect. The biological response to prednisone at low doses (≤7.5 mg/day prednisone or equivalent), is not necessarily predictable in inducing and sustaining an anti-inflammatory effect in RA [29]. If we had used larger doses of prednisone in the study, for example 40mg, we would have undoubtedly seen more consistent time-responses but this would have weakened the impact of the study as it would not have permitted a demonstration of the sensitivity of US to detect change.

The Long STA endpoint performed especially well in the current study. Our previous investigations of HFUS gray-scale ST have shown inferiority to power Doppler vascularity in detecting a treatment effect with respect to the kinetics and the extent of change [27,30]. However, those studies measured synovial thickening semi-quantitatively in the transverse plane only. Semi-quantitative indices may constrain the detection of change in joints if synovial thickening greatly exceeds the largest score by delivering static scores when genuine reduction in synovial thickening can be detected quantitatively. The greater area afforded by the longitudinal versus the transverse view may have also benefited the registration of a treatment effect by the Long STA endpoint. The data in the current study support these theories: semi-quantitative measures of synovial thickening had smaller effect sizes than quantitative measures (the only exception was Day 2, panel B); transverse measures of synovial thickening had smaller effect sizes than longitudinal measures (the only exception was Day 2, Panel A; Figure 2). The treatment effect was less at Day 2 and, therefore, these factors would have had less influence at this early time point.

Most US studies have investigated reliability on a joint by joint basis. Few have assessed reliability of a summation of scores for a selected group of joints. Naredo *et al*. assessed within scan intra-reader reliability with a resultant excellent ICC value of 0.99 [10] for summated 4-point semi-quantitative PDUS imaging of 28 joints, called the 'overall US joint index for power Doppler signal'. Backhaus *et al*. [31] developed a composite US score called the 'German US7 score'. They measured HFUS synovitis and PD synovitis using 4-point semi-quantitative scales in seven joints and the within scan inter-reader reliability kappa value was 0.6. Arguably the most robust measure of reliability is the 'parallel scan inter-reader' (included in our study) because it is a comparison between two ultrasonographer-readers scanning the same patient. The images are read independently, as might be the case in multi-site clinical trials using the same model of US machine and settings. As expected the overall reproducibility for parallel scan inter-reader reliability was lower than within scan inter-reader reliability; the difference between these two methods most likely representing the loss of concordance due to image acquisition. A similar observation was reported by Kamishima *et al*. [32]. Despite this shortfall, in the current study good agreement was observed for the overall parallel scan inter-reader reliability. The overall parallel scan intra-reader reliability was strongest demonstrating the potential advantage of one ultrasonographer acquiring and reading the scans at a single site.

Quantitative ultrasonographic measures of synovitis demonstrated better overall reliability than semi-quantitative measures although the difference was not statistically significant. Therefore, within future studies there may still be a place for more time-consuming measures of synovitis, by computationally quantifying pixel counts, but quicker semi-quantitative scales may be an acceptable substitute. We observed that power Doppler measures of synovitis were significantly more reproducible than gray-scale measures of synovitis and we advocate that future US studies include power Doppler vascularity endpoints to deliver optimum reliability.

The dimensions of the transducers available for use in this study may have been a limitation resulting in weak inter-reader reliability (within-scan or parallel scan) for the 10MCP Trans STi and the 10MCP Trans STA. Because of the broad width of the transducer relative to the deepest point of the triangular structure (which is a narrow precise location), more than one hyperechoic line, representing bone, is often observed on the saved gray-scale image. Therefore, MS and SK may have chosen different ROIs depending on which line was selected to represent the lower border of the triangular structure, even though, from the beginning of the study, there was a consensus to use the lowest hyperechoic line.

Another limitation of our study is that the two prednisone doses were trialed in series rather than in parallel and, therefore, although comparisons can be made between treatment groups, firm conclusions are hampered. This is especially relevant when attempting to comment on the dose-response of the US endpoints to prednisone.

Due to the time constraints of scanning we restricted our US evaluation to the dorsum of the MCPJs. It may have been valuable to have assessed endpoints derived from imaging over the palmar surface also.

## Conclusions

Our study confirms that ultrasonographic imaging of MCPJs could be used as an early and reliable indicator of a therapeutic response to a new treatment intervention in RA early phase clinical trials with small patient cohorts over a two-week test period and decrease the time-to-decision for progressing clinical development. By addressing the issues surrounding the reliability of US to measure synovitis objectively this study brings us closer to approving this tool as a recognized endpoint for confirming treatment effect in RA clinical trials. The semi-quantitative US endpoints demonstrated in this study, whether used alone or together with clinical measures as composite endpoints, could be used in centers not possessing quantitative analysis tools. The study also illustrates the potential utility of US to stratify patient selection by detecting those with potentially reversible baseline joint inflammation, an important consideration given concerns about bias introduced in trials by recruitment of patients with equivocal clinical swelling. Moreover, composite endpoints have the potential to further reduce patient numbers and study duration in early phase trials.

## Abbreviations

ACR: American College of Rheumatology; AE: adverse event; B & L: St Bartholomew's and the London National Health System Trust; CASS: Computerized Allocation Schedule System; CRP: C-reactive protein; DAS28: Disease Activity Score in 28 Joints; DMARDs: disease modifying anti-rheumatic drugs; HFUS: high-frequency ultrasonography; ICC: intra-class correlation coefficients; KIR: Kennedy Institute of Rheumatology; MCPJ: metacarpophalangeal joint; MRI: magnetic resonance imaging; PD: Power Doppler; PDA; Power Doppler Area; PDUS: Power Doppler ultrasonography; ROI: region of interest; STA: synovial thickness area; STi: synovial thickness index; VASCi: vascularity index.

## Competing interests

This study was supported by Merck Sharp & Dohme which was the study sponsor. Drs. Malice, Dardzinski, Cummings, Beals and Smugar, Mr. Bolognese, and Ms. Cheng are or were employees of Merck Sharp & Dohme, Corp.; and Drs. Malice, Beals, Dardzinski and Smugar, and Mr. Bolognese, and Ms. Cheng own stock and/or hold stock options in the company. Dr. Taylor has received research grant support from Merck. Barts and The London School of Medicine received financial support from Merck for the trial. Drs. Pitzalis, Seymour, Kelly and Dooley, and Ms. McClinton and Ms. Fox report no competing interests.

## Authors' contributions

MWS was involved in the design of the study, acquisition, analysis and interpretation of data, drafting and final approval of the manuscript. SK was involved in the design of the study, acquisition, analysis and interpretation of data, drafting and final approval of the manuscript.

CRB was involved in the conception and design of the study, analysis and interpretation of data and drafting and final approval of the manuscript. M-PM was involved in statistical analysis, interpretation of the data and final approval of the manuscript. JAB was involved in the design of the study, analysis and interpretation of the data and final approval of the manuscript. BJD was involved in the administrative support, analysis and interpretation of the data and drafting and final approval of the manuscript. ASC was involved in the administrative support, analysis and interpretation of the data and drafting and final approval of the manuscript. CEC was involved in the administrative support, analysis and interpretation of the data and drafting and final approval of the manuscript. SSS was involved in the statistical analysis, analysis and interpretation of the data and drafting and final approval of the manuscript. CMcC was involved in the administrative support, data acquisition and final approval of the manuscript. AF was involved in the administrative support, data acquisition and final approval of the manuscript. WD was involved in the interpretation and analysis of the data and final approval of the manuscript. CP was involved in the conception and design of the study, interpretation and analysis of the data, drafting and final approval of the manuscript. PCT was involved in the conception and design of the study, interpretation and analysis of the data, drafting and final approval of the manuscript.

All authors read and approved the manuscript for publication. Sabrina Wan, Jennifer Pawlowski, Belma Dogdas and Craig Fancourt were involved with at least one of the following, but did not meet all criteria for authorship: data analysis, administrative support, and software programming for the quantitative analyses.
